# Supplementary material for: Free Energy Projective Simulation (FEPS): Active inference with interpretability
Source: PLoS One. 2025 Sep 4;20(9):e0331047. doi: 10.1371/journal.pone.0331047 (PMC12410762; doi:10.1371/journal.pone.0331047)
Supplement: S1 Appendix — (PDF) [file pone.0331047.s001.pdf]

S1 Appendix: Table of hyperparameters for the numerical analysis.

We summarized the parameters we have used and tested in the numerical analysis of FEPS agents for the delayed reward task, as well as the navigation task in the table below.

Parameters	Skinner box	Navigation
N_{agents}	100	30
N_{clones}	2	3
N_{episodes}	4 000	40 000
Length episodes	80	
Forgetting rate γ	0.001	
Reward scale R	3	
scaling parameter $\zeta_{\text{exploration}}$	{0}	{-3, -1, 0, 1, 3}
scaling parameter ζ_{task}	-1	-3
p*	0.99	
N_{pref}	1	
Prediction horizon T_h	2	3
