# Supplementary material for: Free Energy Projective Simulation (FEPS): Active inference with interpretability
Source: PLoS One. 2025 Sep 4;20(9):e0331047. doi: 10.1371/journal.pone.0331047 (PMC12410762; doi:10.1371/journal.pone.0331047)
Supplement: S2 Appendix — (PDF) [file pone.0331047.s002.pdf]

S2 Appendix: Derivation of the expected free energy in the exploration phase

During the exploration phase, the preference distribution is designed to encourage the agent to seek the relevant information to complete its model. The preferences are equal to the world model marginalized over actions:

$$\mathrm{pref}(S_{t+1}, B_{t+1}|b_t) = \sum_a \pi(a|b_t)\, p(B_{t+1}|b_t, a)\, p(S_{t+1}|B_{t+1}). \tag{1}$$

Plugging this result in the expected free energy in Eq. (4), one obtains:

$$\begin{aligned}
\mathcal{G}_{b_t}[a_t] =& \mathbb{E}_{b_{t+1},s_{t+1}\sim p(B_{t+1},S_{t+1}|b_t,a_t)}[\log p(b_{t+1}|b_t,a_t) - \log \mathrm{pref}(s_{t+1}, b_{t+1}|b_t, a_t)] && (2)\\
=& \sum_{b_{t+1},s_{t+1}} p(b_{t+1}|b_t,a_t)\, p(s_{t+1}|b_{t+1}) && (3)\\
& \left[\log p(b_{t+1}|b_t,a_t) - \log \sum_a \pi(a|b_t)\, p(b_{t+1}|b_t,a)\, p(s_{t+1}|b_{t+1})\right] && (4)\\
=& \sum_{b_{t+1},s_{t+1}} p(b_{t+1}|b_t,a_t)\, p(s_{t+1}|b_{t+1}) && (5)\\
& \left[\log p(b_{t+1}|b_t,a_t) - \log \sum_a \pi(a|b_t)\, p(b_{t+1}|b_t,a) - \log p(s_{t+1}|b_{t+1})\right] && (6)\\
=& \sum_{b_{t+1}} p(b_{t+1}|b_t,a_t) \left[-\sum_s p(s_{t+1}|b_{t+1}) \log p(s_{t+1}|b_{t+1})\right] && (7)\\
& + \sum_{b_{t+1}} p(b_{t+1}|b_t,a_t) \left[\log p(b_{t+1}|b_t,a_t) - \log \sum_a \pi(a|b_t)\, p(b_{t+1}|b_t,a)\right].
\end{aligned}$$

In the second line, we replace the preference distribution by its definition with respect to the world model, and the additivity rule of the logarithm is used to move to the third. In the fourth fourth line, we separated the expectation values over B_{t+1} and S_{t+1} by noticing that for two random variables X, Y, $\mathbb{E}_{x\sim p(X)}[f(Y=y)] = f(y)$ by the normalization constraint on probability distributions. As a result, terms are grouped by dependency, where in particular, the second term has become independent of sensory states. The first term is the conditional entropy of sensory states, conditioned on belief states. For some belief state b_{t+1}, and when the world model is clone-structured, the likelihood is a delta function: $p(s_{t+1}|b_{t+1}) = \delta_{s_{t+1},s(b_{t+1})}$ where $s(b_{t+1})$ designates the observation b_{t+1} is a clone of. As a result, when the sensory states match, the logarithm vanishes, and otherwise, it is multiplied by zero. Therefore, the entropy over observations cancels by design. The remaining term reduces to the Kullback-Leibler divergence between the transition function and the transition function marginalized over actions:

$$\begin{aligned}
\mathcal{G}_{b_t}[a_t] &= \mathbb{D}_{KL}\left[p(B_{t+1}|b_t,a_t)||\sum_a \pi(a|b_t)\, p(B_{t+1}|b_t,a)\right] && (8)\\
&= \mathbb{D}_{KL}\left[p(B_{t+1}|b_t,a_t)||p(B_{t+1}|b_t)\right] && (9)\\
&= \mathbf{IG}(B_{t+1}, A_t = a_t), && (10)
\end{aligned}$$

where $p(B_t|b_t)$ is the marginal of the transition function over actions, and in the last line, $\mathbf{IG}(X|Y=y)$ is the information gain about X from knowing the value of $Y = y$.
