# Supplementary material for: Free Energy Projective Simulation (FEPS): Active inference with interpretability
Source: PLoS One. 2025 Sep 4;20(9):e0331047. doi: 10.1371/journal.pone.0331047 (PMC12410762; doi:10.1371/journal.pone.0331047)
Supplement: S3 Appendix — (PDF) [file pone.0331047.s003.pdf]

S3 Appendix. Limits of the EFE

In this section, we derive, under certain assumptions that we justify with our numerical simulations, a limit for the expected free energy for environments with deterministic transitions, which include the timed response and navigation tasks. Formally, this means that the world model has been fully trained and the transition function yields perfect prediction accuracy over sensory states given the correct belief state is known. Equivalently, the variational free energy is minimized, and the posterior and prior distributions for the transition functions stay essentially identical after each update, i.e. $\exists \epsilon \in \mathbb{R}$, such that $\mathbb{D}_{\mathrm{KL}}[q_\phi(B_t|b_{t-1}, a_{t-1})||p(B_t|b_{t-1}, a_{t-1})] < \epsilon$, where $\epsilon > 0$ can come arbitrarily close to 0. Then, considering the expression for the VFE in Eq. (2), we see that the first term can be neglected and the second term simplifies to $-\sum_{b_t} p(b_t|b_{t-1}, a_{t-1}) \log p(s_t^{\mathrm{env}}|b_t)$, where we used the fact that the Kullback-Leibler divergence cancels out if and only if the two distributions are equal, that is, $q_\phi(B_t|b_{t-1}, a_{t-1}) = p(B_t|b_{t-1}, a_{t-1})$. In this limit, a good approximation of the VFE is: $\mathcal{F} = -\sum_{b_t} p(b_t|b_{t-1}, a_{t-1}) \log p(s_t^{\mathrm{env}}|b_t) = 0$ for any observation received from the environment. Furthermore, our assumption of a perfect world model implies that each belief state b is associated to exactly one hidden state e in the (deterministic) environment. That means that when the agent is in belief state b, the environment is in hidden state e. In these conditions, we say that b *represents* e. As a result, the transition function has the following form:

$$p(b_t|b_{t-1}, a_{t-1}) = \begin{cases} x_b & \text{if } s(b_t) = s_t^{\mathrm{env}} \text{ and } b_t \text{ represents a hidden state } e_t \text{ that emitted } s_t^{\mathrm{env}} \\ 0 & \text{otherwise.} \end{cases} \tag{1}$$

such that $x_b \in [0, 1]$ and the sum of probabilities for all belief states that represent the same hidden state e_t sum up to 1: $\sum_b x_b = 1$. In principle, the values for the x_b can be arbitrary, and depend on the individual models the agents collapse onto after training. We call *children* of b_t and a_t the set of all belief states that can be reached with non-zero probability from b_t with action a_t: $ch(b_t, a_t) = \{b_{t+1}|p(b_{t+1}|b_t, a_t) > 0\}$. Similarly, we call $e(b_t, a_t)$ the hidden state in the environment that results from applying a_t from b_t. $e(b_t, a_t) = e_{t+1}$ designates the fact that starting from belief state b_t and under action a_t, the environment will transition to e_{t+1} in the next step.

Two asymptotic configurations stood out during the numerical simulations: (1) the agent adopts a distributed representation of the hidden state, and any belief state participating in it can be sampled with close to uniform probability (that we idealize to be exactly uniform in the derivation), or (2) reinforcements of individual edges in the models led to the adoption of a single belief state to fully represent the hidden state while ignoring the other clones associated with zero probability. A belief state represents at most one hidden state. In order to accommodate the first possibility, we define the set:

$$\mathcal{D}_{b_t, e_{t+1}} = \{b_{t+1}|\exists a \in \mathcal{A},\ p(b_{t+1}|b_t, a) > 0 \text{ and } b_{t+1} \text{ represents } e_{t+1} \text{ only}\} \tag{2}$$

the set of all belief states that can be transitioned to and that uniquely represent the hidden state $e(b_t, a_t)$. Furthermore, we require that belief states $b_{t+1}, b'_{t+1} \in \mathcal{D}_{b_t, e_{t+1}}$ are degenerate, that is, $\forall a \in \mathcal{A},\ p(b_{t+1}|b_t, a) = p(b'_{t+1}|b_t, a)$. The size $|\mathcal{D}_{b_t,e}|$ of the set a belief state belongs to is called its *degeneracy* and $\forall b \in \mathcal{D}_{b_t, e(b_t, a_t)},\ p(b|b_t, a_t) = 1/|\mathcal{D}_{b_t, e(b_t, a_t)}|$. As a result, the world model becomes:

$$p(b_{t+1}, s_{t+1}|b_t, a_t) = \frac{\delta_{b_{t+1} \in ch(b_t, a_t)}}{|\mathcal{D}_{b_t, e(b_t, a_t)}|} \delta_{s_{t+1}, s(b_{t+1})}, \tag{3}$$

where as in the main text, $\delta_{b_{t+1} \in ch(b_t, a_t)}$ equals 1 if b_{t+1} is a child of b_t and a_t and 0 otherwise, and $s(b_{t+1})$ is the sensory state that b_{t+1} is a clone of, in contrast to s_{t+1}, the value that the sensory state can take. Another important consequence is that if two actions $a, a' \in \mathcal{A}$ lead to the same hidden state in the environment, that is, $e_{t+1} = e(b_t, a) = e(b_t, a')$, then the children of b_t and a, and b_t and a', respectively, are the same: $ch(b_t, a) = ch(b_t, a')$. Conversely, if $e(b_t, a) \neq e(b_t, a')$, then the two sets have no belief state in common: $ch(b_t, a) \cap ch(b_t, a') = \emptyset$. Therefore, the following holds for any two actions $a, a' \in \mathcal{A}$:

$$\exists b \in \mathcal{B} \text{ such that } b \in ch(b_t, a) \text{ and } b \in ch(b_t, a') \iff ch(b_t, a) = ch(b_t, a') \tag{4}$$

Using Eq. (3) for the world model, the new expression for the expected free energy (Eq. (4)in the main text) is:

$$\mathcal{G}_{b_t}[a_t] = \sum_{b_{t+1}, s_{t+1}} \frac{\delta_{b_{t+1} \in ch(b_t, a_t)}}{|\mathcal{D}_{b_t, e(b_t, a_t)}|} \delta_{s_{t+1}, s(b_{t+1})} \log \left(\frac{\delta_{s_{t+1}, s(b_{t+1})}/|\mathcal{D}_{b_t, e(b_t, a_t)}|}{\mathrm{pref}(b_{t+1}, s_{t+1}|b_t)} \right) \tag{5}$$

$$= \sum_{b_{t+1} \in ch(b_t, a_t), s_{t+1}} \frac{1}{|\mathcal{D}_{b_t, e(b_t, a_t)}|} \delta_{s_{t+1}, s(b_{t+1})} \log \left(\frac{1/|\mathcal{D}_{b_t, e(b_t, a_t)}|}{\mathrm{pref}(b_{t+1}, s_{t+1}|b_t)} \right). \tag{6}$$

Exploration phase: During the exploration phase, the preferences of the agents are set as the marginal of their world model over actions:

$$\text{pref}(b_{t+1}, s_{t+1}|b_t) = \sum_a p(s_{t+1}|b_{t+1})\, p(b_{t+1}|b_t, a)\, \pi(a|b_t) \tag{7}$$

$$= \sum_a \delta_{s_{t+1}, s(b_{t+1})} \frac{\delta_{b_{t+1} \in \text{ch}(b_t, a)}}{|\mathcal{D}_{b_t, e(b_t, a)}|} \pi(a|b_t) \tag{8}$$

and the EFE is determined by the final policy:

$$\mathcal{G}_{b_t}[a_t] = \sum_{b_{t+1} \in \text{ch}(b_t, a_t)} \frac{1}{|\mathcal{D}_{b_t, e(b_t, a)}|} \log \left(\sum_{a|e(b_t,a)=e(b_t,a_t)} \frac{1/|\mathcal{D}_{b_t, e(b_t, a_t)}|}{\pi(a|b_t) \cdot 1/|\mathcal{D}_{b_t, e(b_t, a)}|} \right), \tag{9}$$

where by Eq. (4), the sum over actions in the logarithm is restricted to those whose children include b_{t+1}, or equivalently, represent the same hidden state and belong to the same set of degenerate states. Therefore, the degeneracies inside the logarithm cancel each other out, and each term in the sum is the same for each degenerate belief state:

$$\mathcal{G}_{b_t}[a_t] = -\log \left(\sum_{a|e(b_t,a)=e(b_t,a_t)} \pi(a|b_t) \right). \tag{10}$$

In order to find the final value the EFE converges to, we must find the fixed point for the policy. This means that the policy $\pi^{(n+1)}$ after the n-th update using the expected free energy is equal to the original policy at step n, $\pi^{(n)} = \pi$:

$$\pi(a_t|b_t) = \pi^{(n)}(a_t|b_t) \tag{11}$$

$$= \pi^{(n+1)}(a_t|b_t) \tag{12}$$

$$= \text{softmax}(\zeta \mathcal{G}_{b_t}[a_t]) \tag{13}$$

$$= \frac{\exp\left(-\zeta \log\left(\sum_{a|e(b_t,a)=e(b_t,a_t)} \pi(a|b_t)\right)\right)}{\sum_{a'} \exp\left(-\zeta \log\left(\sum_{a''|e(b_t,a'')=e(b_t,a')} \pi(a''|b_t)\right)\right)} \tag{14}$$

$$= \frac{\left(\sum_{a|e(b_t,a)=e(b_t,a_t)} \pi(a|b_t)\right)^{-\zeta}}{\sum_{a'} \left(\sum_{a''|e(b_t,a'')=e(b_t,a')} \pi(a''|b_t)\right)^{-\zeta}} \tag{15}$$

where we have used $\exp(y \log x) = (\exp(\log x))^y = x^y$ to get the last line and b'_{t+1} in the denominator is a belief state that can be reached from b_t with action $a' \in \mathcal{A}$.

To proceed further, we introduce the following notations:

- $\mathcal{E}_{b_t} := \{e^k_{t+1} \mid \exists a \in \mathcal{A} \text{ such that } e(b_t, a) = e^k_{t+1}\}$, the set of hidden states that can be reached from the belief state b_t;
- $\mathcal{A}_{b_t,k} := \{a \mid e(b_t, a) = e^k_{t+1},\ a \in \mathcal{A}\}$, the set of actions that induce a transition to the k-th hidden state e^k_{t+1} in $\mathcal{E}_{b_t}$. $|\mathcal{A}_{b_t,k}|$ is the number of elements in $\mathcal{A}_{b_t,k}$. It has at least one element;
- $\forall e^k_{t+1} \in \mathcal{E}_{b_t}, \forall a \in \mathcal{A}_{b_t,k},\ \pi_{b_t,k} = \pi(a|b_t)$ denotes the probability of taking any action a that will result in the k-th hidden state e^k_{t+1} in $\mathcal{E}_{b_t}$. All actions that result in the same hidden state e^k_{t+1} have the same probability in the policy, as can be seen in Eq. (15).

We rewrite Eq. (15) with this notations, assuming $a_t \in \mathcal{A}_{b_t,k}$:

$$\pi(a_t|b_t) = \pi_{b_t,k} \tag{16}$$

$$= \frac{(|\mathcal{A}_{b_t,k}|\pi_{b_t,k})^{-\zeta}}{\sum_l |\mathcal{A}_{b_t,l}|\ (|\mathcal{A}_{b_t,l}|\pi_{b_t,l})^{-\zeta}} \tag{17}$$

$$= \frac{|\mathcal{A}_{b_t,k}|^{-\zeta/(1+\zeta)}}{\left(\sum_l |\mathcal{A}_{b_t,l}|\ (|\mathcal{A}_{b_t,l}|\pi_{b_t,l})^{-\zeta}\right)^{1/(1+\zeta)}} \tag{18}$$

$$= N \times |\mathcal{A}_{b_t,k}|^{-\zeta/(1+\zeta)} \tag{19}$$

where from the second to third line, we used that for $x \neq 0$, $x = yx^{\alpha} \iff x^{1-\alpha} = y$, and N is the normalization factor in the second to last line. N does not depend on the action considered and it normalizes the policy to 1:

$$\sum_k |\mathcal{A}_{b_t,k}| \, \pi_{b_t,k} = N \times \sum_k |\mathcal{A}_{b_t,k}|^{1/(1+\zeta)} = 1. \tag{20}$$

Therefore, we find $N = 1/\sum_k |\mathcal{A}_{b_t,k}|^{1/(1+\zeta)}$. At the end of a successful training in a deterministic environment, the policy converges to:

$$\pi_{b_t,k} = \frac{|\mathcal{A}_{b_t,k}|^{-\zeta/(1+\zeta)}}{\sum_l |\mathcal{A}_{b_t,l}|^{1/(1+\zeta)}}. \tag{21}$$

Finally, during the exploration phase, once the world model perfectly represents the environment, the expected free energy in Eq. (10) related to taking an action $a_t \in \mathcal{A}_{b_t,k}$ converges to:

$$\mathcal{G}_{b_t}[a_t] = -\log\left(\sum_{a' \in \mathcal{A}_{b_t,k}} \frac{|\mathcal{A}_{b_t,k}|^{-\zeta/(1+\zeta)}}{\sum_l |\mathcal{A}_{b_t,l}|^{1/(1+\zeta)}}\right) \tag{22}$$

$$= -\log\left(\frac{|\mathcal{A}_{b_t,k}|^{1/(1+\zeta)}}{\sum_l |\mathcal{A}_{b_t,l}|^{1/(1+\zeta)}}\right). \tag{23}$$

In Figs 4 and 5, we average this value over actions and previous belief states, since it would otherwise depend on individual deliberations of the agents.
